# Spleen enlargement assessment using computed tomography: which coefficient correlates the strongest with the real volume of the spleen?

**DOI:** 10.1007/s00261-018-1500-9

**Published:** 2018-02-19

**Authors:** Iwona Kucybała, Szymon Ciuk, Justyna Tęczar

**Affiliations:** 1 Department of Radiology, Jagiellonian University Medical College, 19 Kopernika Street, 31-501 Kraków, Poland; 2 Department of General and Interventional Radiology and Neuroradiology, Wroclaw Medical University, 213 Borowska Street, 50-556 Wrocław, Poland

**Keywords:** Spleen, Splenomegaly, Spleen volume, Splenic index, Computed tomography

## Abstract

**Purpose:**

The aim of the study was to find which linear measurements, field and volume coefficients correlate the best with real volume of the spleen and can be further used for identification of splenomegaly.

**Methods:**

Abdominal computed tomography (CT) examinations of 264 patients were retrospectively analyzed in terms of maximal length, maximal thickness, hilum thickness, maximal height, vertical height and estimated height. Spleen volume was manually measured in Vitrea software. Two- and three-dimensional coefficients were calculated through proper mathematical formulas from linear measurements. Splenomegaly cut-off: 314.5 cm^3^. Data were analyzed with use of Pearson correlation and *χ*^2^ test with statistical significance at *p* < 0.05.

**Results:**

For single measurements, the correlation with real spleen volume was the strongest for maximal height (*r* = 0.804; *p* < 0.05). Among two-dimensional indexes, multiplication product of maximal length and vertical height reached the highest level of correlation with spleen volume (*r* = 0.923; *p* < 0.05) and had the highest sensitivity and specificity (94.3% and 93.0%, respectively) for splenomegaly detection (threshold 115 cm^2^). In case of three-dimensional ones, the coefficient calculated from maximal length, vertical height and hilum thickness established the strongest link with spleen volume (*r* = 0.956; *p* < 0.05).

**Conclusions:**

Coefficient calculated from maximal length, vertical height and hilum thickness correlates the strongest with spleen volume and can be utilized for monitoring of spleen volume instead of obsolete splenic index. The most suitable for quick splenomegaly screening is two-dimensional coefficient (maximal length × vertical height), with the cut-off 115 cm^2^.

Splenomegaly is a common clinical pathological finding of various aetiologies, including congestive, excessive antigenic stimulation, destruction of abnormal blood cells and neoplastic infiltration. It may be detected in symptomatic and coincidentally, in asymptomatic patients. Thus, appropriate evaluation of the spleen size seems to be necessary in order to initiate a diagnostic process, take proper therapeutic decisions and follow treatment effect in definite cases [1, 2].

Primarily, the clinical diagnosis of splenomegaly is defined as the organ palpable below the costal margin during physical examination [3]. However, the recognition of the spleen enlargement by means of imaging methods such as ultrasonography or computed tomography was proved to be more sensitive [4]. Currently, computed tomography (CT) is considered as a highly accurate and reproducible diagnostic tool for spleen morphology assessment, since it allows to thoroughly evaluate the spleen on multiple intersections, and is widely available and relatively cheap [5].

There have been multiple approaches for a precise evaluation of spleen size basing on CT examination. To this end, individual linear measurements, multidimensional coefficients as well as volume measurements have been applied. These include a popular, but rather obsolete formula called splenic index—multiplication product of maximal length, estimated height and maximal thickness of the spleen with the upper normality limit for splenomegaly set at 480 [6].

Clinical routine requires simultaneously reliable and practical solutions. At present, clear and unequivocal guidelines for splenomegaly assessment in CT are lacking. Therefore, the aim of this study was to find which linear measurements and two-dimensional and three-dimensional coefficients correlate the best with real volume of the spleen and can be further used for determination of splenomegaly.

## Materials and methods

### Study population

We included into the study and retrospectively analyzed 264 abdominal CT examinations, which were carried out at the Department of Diagnostic Imaging of the University Hospital (Szpital Uniwersytecki) in Cracow in the period between September and December 2016. Mean age of examined patients was 58.8 ± 15.5 years, age ranged between 16 and 91 years. 48.5% (*n* = 128) of the patients were female and 51.5% (*n* = 136) male. Patients included into the study were referred to the Department of Diagnostic Imaging to undergo abdominal CT due to various clinical symptoms related to the abdominal region. The exclusion criteria were spleen injury, multi-organ injury, spleen focal lesion, previous splenectomy and any condition that would affect the position of the spleen.

### Technical parameters

All of the scans were performed with use of an 80-row helical CT scanner Toshiba Aquilion PRIME 80 (Toshiba America Medical System, Irvine, CA), in an abdominal protocol, scan parameters: 120 kV, 385 mAs; rotation time 500 ms; slice thickness 2.0 mm, interval 1.6 mm, to acquire axial images of the abdominal region. Coronal sections were obtained using multiplanar reconstructions (iterative reconstruction method), parameters: slice thickness 2.0 mm, interval 2.0 mm.

### Measurements methodology

Linear measurements and volume of the spleen parenchyma (*V*_spl_) were assessed with the use of Vitrea Software 6.7.1 (Vital Images Inc., Minnetonka, MN, USA). All linear measurements were done in millimeters, while volume of the spleen was calculated in cm^3^. All values of measurements were presented as the value ± standard deviation.

**Fig. 1:**
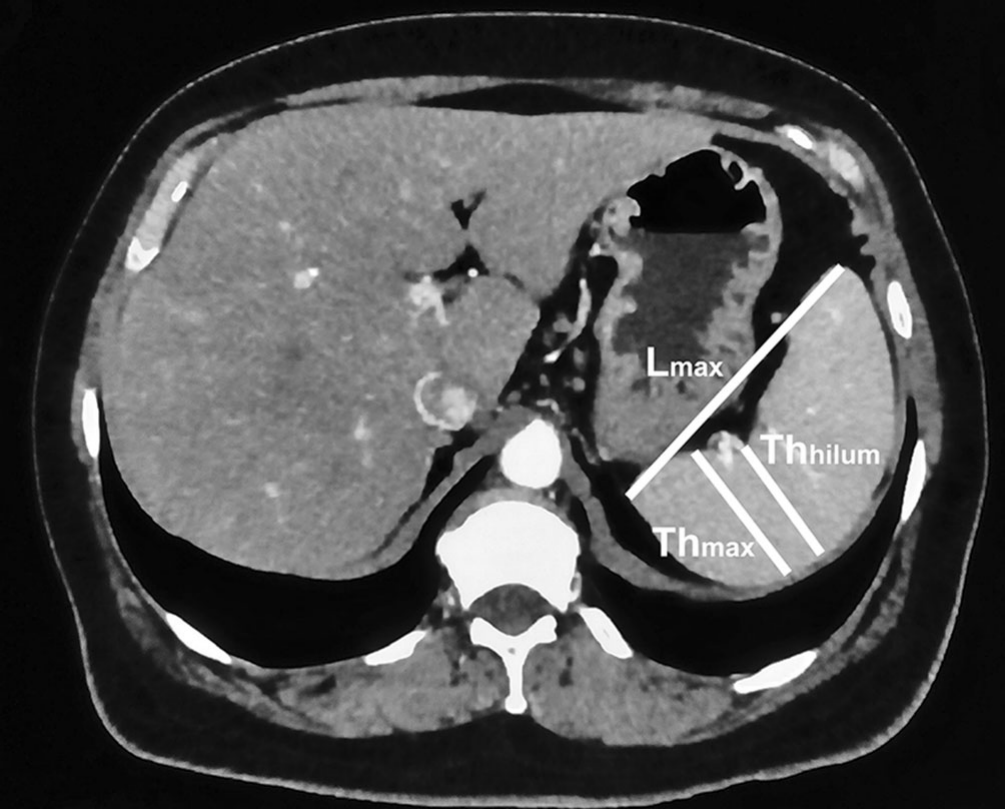
The method for measuring of maximal length (*L*_max_), maximal thickness (Th_max_) and hilum thickness (Th_hilum_)

Linear dimensions of the spleen measured in the axial section were (Fig. 1):

- maximal length (*L*_max_)—the longest dimension between poles of the spleen in this section,
- maximal thickness (Th_max_)—the widest dimension perpendicular to the long axis of the spleen,
- hilum thickness (Th_hilum_)—dimension measured in central part of the hilum perpendicularly to the long axis of the spleen.

**Fig. 2:**
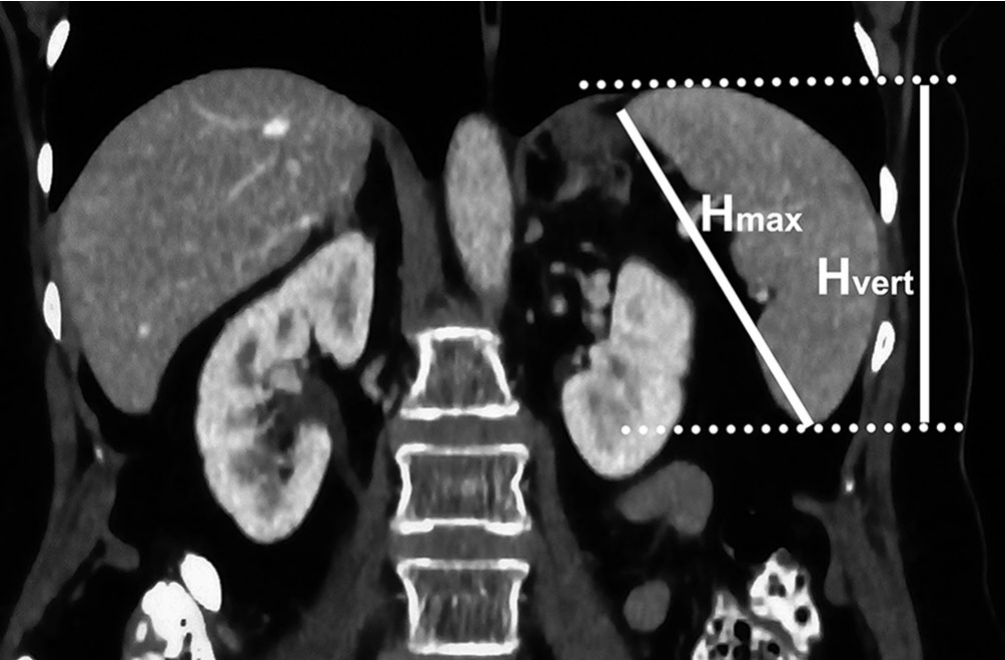
Dimensions measured on the coronal plane—maximal height (*H*_max_) and vertical height (*H*_vert_)

In the coronal section (Fig. 2):

- maximal height (*H*_max_)—the longest dimension between poles of the spleen in this section,
- vertical height (*H*_vert_)—the longest vertical dimension between cranial and caudal borders of the spleen.

Additionally, estimated height of the spleen (*H*_est_) was assessed as the number of axial scans with the spleen visible multiplied by the slice thickness.

Two-dimensional (2D) and three-dimensional (3D) coefficients were calculated as the product of multiplication of respectively two (length × thickness, length × height, height × thickness) or three various dimensions (length × thickness × height). In our study, 11 two-dimensional and 6 three-dimensional coefficients were counted.

**Fig. 3:**
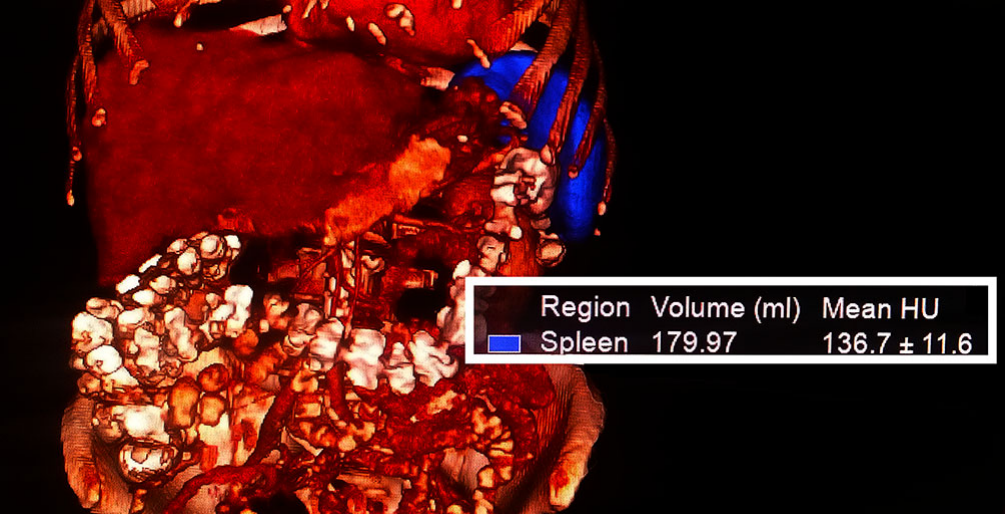
Three-dimensional reconstruction of the spleen and the calculation of its volume

The real spleen volume was measured manually with the Volume Measurement Tool by defining the frontiers of the spleen on the surface of fibrous capsule (in a soft tissue window) in the coronal section. Subsequently, the volume was calculated by the software and three-dimensional reconstruction of the organ was obtained (Fig. 3).

All measurements of the spleen were evaluated by two independent examiners simultaneously and the mean of two separate measurements of the identical parameter was classified as the final value. All significant discrepancies in measurements between examiners were analyzed and assessed one more time in the presence of a third examiner.

### Statistical analysis

Statistical analysis was performed in Statistica 12.5 software (StatSoft Inc., Tulsa, OK, USA). Statistical significance was set at *p* < 0.05. Linear Pearson’s correlation was used to assess the strength of correlation between dimensions along with coefficients and volume of the spleen.

Cut-off points for detection of splenomegaly for linear measurements along with two-dimensional and three-dimensional coefficients were calculated in each case from equations of linear regression calculated by the software. Value of 314.5 cm^3^ as volumetric threshold of splenomegaly, according to Prassopoulos et al. study [6], was substituted into specific linear regression equation and the product of this calculation was considered as the splenomegaly cut-off value of certain parameter. In cases of linear dimensions, all results were rounded up or down to the nearest half while products of two-dimensional and three-dimensional coefficients were rounded up or down to the nearest integer. In the next step, Chi square test, sensitivity, specificity, positive predictive value (PPV) and negative predictive value (NPV) were applied so as to evaluate the capability of calculated threshold to detect splenomegaly according to volumetric criterion and to assess its effectiveness.

## Results

13.3% (*n* = 35) of patients in the study had splenomegaly according to previously mentioned volumetric criterion. Reasons of spleen enlargement in our study were liver cirrhosis, Hodgkin lymphoma, chronic lymphoid leukemia (CLL), hemolytic anemia and acquired immune deficiency syndrome (AIDS). Mean volume of the spleen were 178.1 ± 64.2 and 481.4 ± 242.0 cm^3^ in groups without and with splenomegaly, respectively. Any statistically significant differences in terms of age and gender between groups with or without splenomegaly were not detected (Table 1).

**Table 1:** Demographic data of groups with or without splenomegaly

	Splenomegaly	Without splenomegaly	*p* value
Age ± SD (years)	55.2 ± 12.6	59.3 ± 15.1	0.120
Females (%)	40.0	49.8	0.281

### Linear measurements

The strongest correlation with splenic volume was detected for maximal height (*r* = 0.804; *p* < 0.05). Splenomegaly threshold for this parameter was 12 cm (equation of linear regression: *V*_spl_ = 6.393 × *H*_max_ + 444.129). Sensitivity, specificity, PPV and NPV values were 71.4, 91.7, 56.8 and 95.5%.

Similar strength of correlation was observed in case of vertical height (*r* = 0.790; *p* < 0.05). Calculated splenomegaly cut-off was 10.5 cm (equation: *V*_spl_ = 4.986 × *H*_vert_ + 210,541). This dimension improved values of sensitivity, specificity, PPV and NPV: 82.9, 91.7, 60.4 and 97.2%, respectively.

Profile of all linear dimensions is shown in Table 2.

**Table 2:** Correlation between linear dimensions and volume of the spleen; calculated splenomegaly cut-offs and their effectiveness in detection of volumetric splenomegaly; in bold—best correlation

Linear dimension	Pearson linear correlation		Splenomegaly cut-off (cm)	*χ* ^2^ *p* value	Sensitivity (%)	Specificity (%)	PPV (%)	NPV (%)
	*p* value	*r*						
Maximal length	*p* < 0.05	0.756	11.5	*p* < 0.05	80.0	90.8	57.1	96.7
Maximal thickness		0.585	7		37.1	90.4	37.1	90.4
Hilum thickness		0.649	4		68.6	76.0	30.4	94.1
**Maximal height**		**0.804**	**12**		**71.4**	**91.7**	**56.8**	**95.5**
Vertical height		0.790	10.5		82.9	91.7	60.4	97.2
Estimated height		0.563	15.5		40.0	91.3	41.2	90.9

### Two-dimensional coefficients

Multiplication product of maximal length and vertical height correlated the strongest with volume of the spleen (*r* = 0.923; *p* < 0.05; equation: *V*_spl_ = 3.514 × (*L*_max_ × *H*_vert_) − 92.464). Calculated splenomegaly threshold: 116 cm^2^. Approximation to 115 cm^2^ did not have any impact on qualitative parameters of effectiveness of the coefficient. Sensitivity, specificity, PPV and NPV values for 115 and 116 cm^2^ were 94.3, 93.0, 67.3 and 99.1%, respectively. Rounding up to 120 cm^2^ caused a slight decline in the value of sensitivity (91.4%) and NPV (98.6%) still remaining at a high level and elevation of specificity (94.3%) and PPV (71.1%).

Details regarding all two-dimensional coefficients are demonstrated in Table 3.

**Table 3:** Correlation between two-dimensional coefficients and volume of the spleen along with splenomegaly thresholds and their effectiveness in detection of volumetric splenomegaly; in bold—best correlation

Multiplied dimensions		Pearson linear correlation		Splenomegaly cut-off (cm^2^)	*χ* ^2^ *p* value	Sensitivity (%)	Specificity
Dimension 1	Dimension 2	*p* value	*r*				
Maximal length	Maximal thickness	*p* < 0.05	0.816	72	*p* < 0.05	65.7	92.6
Maximal length	Hilum thickness		0.853	47		68.6	90.8
Maximal length	Maximal height		0.912	132		85.7	90.4
Maximal height	Maximal thickness		0.821	75		65.7	90.0
Maximal height	Hilum thickness		0.851	49		65.7	90.4
**Maximal length**	**Vertical height**		**0.923**	**115**		**94.3**	**93.0**
				**116**		**94.3**	**93.0**
				**120**		**91.4**	**94.3**
Maximal length	Estimated height		0.766	161		71.4	93.9
Maximal thickness	Vertical height		0.864	65		68.6	93.9
Maximal thickness	Estimated height		0.726	91		54.3	92.1
Hilum thickness	Vertical height		0.885	43		71.4	92.1
Hilum thickness	Estimated height		0.757	59		54.3	92.6

### Three-dimensional coefficients

The strongest correlation with the spleen volume was discovered for a coefficient calculated by multiplication of maximal length, vertical height and hilum thickness (*r* = 0.956; *p* < 0.05; linear regression equation: *V*_spl_ = 0.601 × (*L*_max_ × *H*_vert_ × Th_hilum_) + 18.889). The cut-off point for splenomegaly was 492 cm^3^. Values of sensitivity, specificity, PPV and NPV were equal for 492 cm^3^ and approximated 490 cm^3^: 82.9%, 95.6%, 74.4% and 97.3%, respectively. Rounding up to 500 cm^3^ had a negative impact on specificity (77.1%) and NPV (96.5%), but slightly increased the level of specificity (96.5%) and PPV (77.1%).

A coefficient counted by multiplication of maximal length, vertical height and maximal thickness had a fine correlation with the spleen volume as well (*r* = 0.946; *p* < 0.05; equation: *V*_spl_ = 0.403 × (*L*_max_ × *H*_vert_ × Th_max_) + 18.889). Additionally, for the threshold at the level of 739 cm^3^, it had improved values of specificity (96.1%) and PPV (76.3%) along with equal sensitivity and NPV to the above-mentioned coefficient. Nonetheless, approximation to 740 cm^3^ alters sensitivity, specificity, PPV and NPV to values 80%, 96.5%, 77.8% and 96.9%, respectively.

The correlation of splenic index (product of multiplication of maximal length, estimated height and maximal thickness) with volume of the spleen was rather poor (*r* = 0.846; *p* < 0.05; equation: *V*_spl_ = 0.256 × (*L*_max_ × *H*_est_ × Th_max_) + 56.023), compared to other three-dimensional coefficients. The calculated cut-off point for that coefficient was 1010 cm^3^. Sensitivity, specificity, PPV and NPV were 62.9, 93.9, 61.1 and 94.3%, respectively.

A detailed list of all parameters of three-dimensional coefficients is included in Table 4.

**Table 4:** Correlation between three-dimensional coefficients and volume of the spleen; calculated splenomegaly cut-offs and their effectiveness in detection of volumetric splenomegaly; in bold—best correlation and in italics—splenic index

Multiplied dimensions			Pearson linear correlation		Splenomegaly cut-off (cm)	*χ* ^2^ *p* value	Sensitivity (%)	Specificity
Dimension 1	Dimension 2	Dimension 3	*p* value	*r*				
Maximal length	Maximal height	Maximal thickness	*p* < 0.05	0.926	840	*p* < 0.05	82.9	93.0
Maximal length	Maximal height	Hilum thickness		0.940	557		68.6	94.8
Maximal length	Vertical height	Maximal thickness		0.946	739		82.9	96.1
					740		80.0	96.5
**Maximal length**	**Vertical height**	**Hilum thickness**		**0.956**	**490**		**82.9**	**95.6**
					**492**		**82.9**	**95.6**
					**500**		**77.1**	**96.5**
*Maximal length*	*Estimated height*	*Maximal thickness*		*0.846*	*1010*		*62.9*	*93.9*
Maximal length	Estimated height	Hilum thickness		0.873	660		62.9	94.3

## Discussion

The use of imaging techniques is recently considered to be the most accurate method of both diagnosis and follow-up in the case of splenomegaly. Literature suggests that CT should identify changing spleen volume with the highest sensitivity and specificity [7], although other imaging techniques such as ultrasonography [8, 9] or magnetic resonance imaging (MRI) [10] can also be used. Additionally, a number of studies proved significant accuracy of the CT in assessment of the spleen, both in pediatric and adult populations [11, 12].

Among all coefficients, three-dimensional ones seem to be the best means of spleen size evaluation, since they represent the highest level of correlation with real volume of the spleen. The highest correlation coefficient was detected for the multiplication product of maximal length, vertical height and hilum thickness of the spleen (*r* = 0.956; *p* < 0.05). Moreover, the coefficient calculated from maximal length, vertical height and maximal thickness had a fine correlation with the spleen volume as well (*r* = 0.946; *p* < 0.05). Thus, they can be valuable for the assessment of changes in the spleen volume in cases in which this parameter is helpful for stating the diagnosis (sarcoidosis, rheumatoid arthritis, lupus, conditions ongoing with portal hypertension), or monitoring the clinical condition, e.g., after spleen embolization, during the treatment of hemolytic anemia, myeloproliferative disorders, lymphoproliferative syndromes, acute leukemia, storage disorders (like Gaucher’s or Neumann–Pick’s disease) [13]. In contrast to above-mentioned coefficients, splenic index happened to have a rather weak correlation with real spleen volume (*r* = 0.846; *p* < 0.05). Lackner et al. introduced the term of splenic index defined as a multiplication product of maximal length, estimated height and maximal thickness of the spleen. Comparing its value set with the use of computed tomography with the one assessed during the autopsy, it was observed that CT have a tendency to underestimate and overestimate this index. As for its limitations, a study was carried out on a relatively small group (118 patients) in comparison to ours (264 patients). Additionally, Lackner et al. study was carried out in the times when CT quality was rather poor in comparison to the technology at present, which could have affected the accuracy of measurements. However, age, race and gender differences in spleen volume were not taken into account in both studies [14]. Since the splenic index is a useful tool for instance in diagnostics of the Hodgkin lymphoma [2] and makes the sensitivity of diagnosis higher, it would be vital to update the coefficient for more clinical accuracy. The threshold indicating splenomegaly calculated for the coefficient with the strongest correlation with spleen volume (maximal length × vertical height × hilum thickness) in our study was very similar to the one suggested in the original study (490 vs. 480 cm^3^, respectively). Nevertheless, the cut-off point for the splenic index calculated in our study (1010 cm^3^) differs considerably from the one suggested by Lackner et al., which makes their coefficient totally unreliable to use.

The two-dimensional coefficient, set as a result of multiplying maximal length and vertical height, represented a high level of correlation with the real volume of the spleen (*r* = 0.923; *p* < 0.05). Moreover, it happened to present the highest sensitivity and specificity (94.3% and 93.0%, respectively), which makes it a great screening tool for radiologists for the quick evaluation of the spleen. With a threshold indicating splenomegaly set at the level of 115 cm^2^, it would help to give a hint for clinicians if they should take a closer look on the spleen and supposedly pay more attention to this organ, and generally, conditions connected with spleen enlargement in terms of the future diagnostic process. Since this coefficient is a combination of measurements done in two sections—axial and coronal, it lowers the chance to overlook the spleen enlargement, in comparison to two-dimensional coefficients based on dimensions measured only in one section. The splenomegaly threshold can be rounded up to 120 cm^2^ (to be easily memorized). Despite still high level of specificity (94.3%), this approximation leads to a decline in sensitivity (91.4%) and consequently, it needs to be considered if such changes are worth establishing.

Considering the linear measurements, maximal height (*r* = 0.804; *p* < 0.05) of the spleen represented the strongest correlation with the volume. This measurement is consistent with the so-called cephalocaudal spleen height, which is used in clinical practice for a quick assessment of the spleen size in CT [15]. Bezerra et al. suggested length (maximal length in our study) and width (our maximal thickness) as the good indicators of spleen enlargement, but they both did not represent the strongest of correlation with the volume (*r* = 0.756 and *r* = 0.585, respectively) [16]. Maximal height parameter is consistent with craniocaudal length of the spleen measured in the ultrasound technique and so is the threshold suggesting splenomegaly for both tools—12 cm [17]. It needs to be remembered that these unidimensional measurements are characterized with a few shortcomings, which derive from the different morphology or localization of the spleen. Moreover, spleen might enlarge in multiple directions, sometimes irregularly and therefore this method of assessment should be reserved for just a quick overview of this organ. However, it is still the least time-consuming method of a brief evaluation.

There are also more complex methods of defining splenomegaly, such as the relationship of the spleen to the left lobe of the liver (ultrasound) [18], or to the left kidney (CT) [16], but they are of no statistical significance or low sensitivity, respectively. Additionally, measurements done with the use of ultrasound are characterized with a low level of repeatability and depend much on the knowledge and experience of a radiologist performing the examination. The shape and spatial orientation of the spleen make it difficult to capture accurate linear measurement and to assess all three dimensions for the most reliable volume calculation. Yetter et al. invented a complicated formula, which includes width, thickness, maximum length and craniocaudal length, which correlates the best to the CT measurements [11]. However, that method of assessment is extremely intricate, requires significant amount of effort and was not useful in clinical practice. Furthermore, Chen et al. suggested the use of MRI in the assessment of spleen volume (in diagnosis of the level of liver cirrhosis) should be reliable, but, in fact, it is a more expensive and time-consuming tool [10]. Due to the multiplicity of sequences, this method should stay reserved for a detailed diagnosis of focal or diffuse lesions, which might look similar in the CT examination [19].

The spleen volume is directly linked with the weight of the organ. It has been known for a long time that spleen weight increases proportionally to the body weight, declines with the age and is generally slightly smaller among women [20]. Thus, for a detailed and thorough examination of the spleen, different thresholds should be established for groups divided according to age, sex and BMI. As well, the number of our patients with splenomegaly could also be the limitation; therefore, a larger, preferably multicenter, study should be also conducted.

It needs to be remembered that the definition of splenomegaly is highly individual and its diagnosis should always be tightly bound with a clinical condition of the patient, rather than treating it as an isolated radiologic diagnosis. However, the coefficients and thresholds can play a vital role in catching the change in spleen size at an early stage. Despite geometrical limitations (actual round shape of the spleen vs coefficients counted as cuboid figures), all coefficients represented a high level of correlation with the real volume of the spleen and can be used as a supportive tool in the daily clinical practice.

## Conclusion

Due to the poor accuracy of splenic index, it should be replaced with:

- three-dimensional coefficient calculated as:
  $${\text{maximal length }} \times {\text{ hilum thickness }} \times {\text{ vertical height,}}$$
  which establishes the strongest correlation with real spleen volume and could be assessed in order to objectively monitor spleen volume.
- or two-dimensional coefficient, resulting from equation:
  $${\text{maximal length }} \times {\text{vertical height,}}$$
  which simultaneously correlates well with the real spleen volume and has the highest values of sensitivity and specificity for the threshold of 115, which makes it a great tool for quick splenomegaly screening in CT examination.

## References

[1] Wu WC, Chiou YY, Hung HH (2012). Prognostic significance of computed tomography scan-derived splenic volume in hepatocellular carcinoma treated with radiofrequency ablation. J Clin Gastroenterol.

[2] Strijk SP, Wagener DJ, Bogman MJ, de Pauw BE, Wobbes T (1985). The spleen in Hodgkin disease: diagnostic value of CT. Radiology.

[3] Brown NF, Marks DJ, Smith PJ, Bloom SL (2011). Splenomegaly. Br J Hosp Med (Lond).

[4] Grover SA, Barkun AN, Sackett DL (1993). The rational clinical examination. Does this patient have splenomegaly?. JAMA.

[5] Rabushka LS, Kawashima A, Fishman EK (1994). Imaging of the spleen: CT with supplemental MR examination. Radiographics.

[6] Prassopoulos P, Daskalogiannaki M, Raissaki M, Hatjidakis A, Gourtsoyiannis N (1997). Determination of normal splenic volume on computed tomography in relation to age, gender and body habitus. Eur Radiol.

[7] Linguraru MG, Sandberg JK, Jones EC, Summers RM (2013). Assessing splenomegaly: automated volumetric analysis of the spleen. Acad Radiol.

[8] Lamb PM, Lund A, Kanagasabay RR, Martin A, Webb JA, Reznek RH (2002). Spleen size: how well do linear ultrasound measurements correlate with three-dimensional CT volume assessments?. Br J Radiol.

[9] Elstein D, Hadas-Halpern I, Azuri Y, Abrahamov A, Bar-Ziv Y, Zimran A (1997). Accuracy of ultrasonography in assessing spleen and liver size in patients with Gaucher disease: comparison to computed tomographic measurements. J Ultrasound Med.

[10] Chen XL, Chen TW, Li ZL (2013). Spleen size measured on enhanced MRI for quantitatively staging liver fibrosis in minipigs. J Magn Reson Imaging.

[11] Yetter EM, Acosta KB, Olson MC, Blundell K (2003). Estimating splenic volume: sonographic measurements correlated with helical CT determination. AJR Am J Roentgenol.

[12] Prassopoulos P, Cavouras D (1994). CT assessment of normal splenic size in children. Acta Radiol.

[13] Ishibashi H, Higuchi N, Shimamura R, Hirata Y, Kudo J, Niho Y (1991). Sonographic assessment and grading of spleen size. J Clin Ultrasound.

[14] Lackner K, Brecht G, Janson R, Scherholz K, Lützeler A, Thurn P (1980). The value of computer tomography in the staging of primary lymph node neoplasms (author’s transl). Rofo.

[15] Linguraru MG, Sandberg JK, Li Z, Pura JA, Summers RM (2009). Atlas-based automated segmentation of spleen and liver using adaptive enhancement estimation. Med Image Comput Comput Assist Interv.

[16] Bezerra AS, D’Ippolito G, Faintuch S, Szejnfeld J, Ahmed M (2005). Determination of splenomegaly by CT: is there a place for a single measurement?. AJR Am J Roentgenol.

[17] Andrews MW (2000). Ultrasound of the spleen. World J Surg.

[18] Cerri GG, Rocha DC, Cerri GG, Prando A (1985). Baço. Ultra-sonografia abdominal (in Portuguese).

[19] Elsayes KM, Narra VR, Mukundan G, Lewis JS, Menias CO, Heiken JP (2005). MR imaging of the spleen: spectrum of abnormalities. Radiographics.

[20] DeLand FH (1970). Normal spleen size. Radiology.

